# Therapeutic Sequences of Systemic Therapy After Atezolizumab Plus Bevacizumab for Hepatocellular Carcinoma: Real‐World Analysis of the IMMUreal Cohort

**DOI:** 10.1111/apt.70090

**Published:** 2025-04-04

**Authors:** Najib Ben Khaled, Valentina Zarka, Bernard Hobeika, Julia Schneider, Monika Rau, Alexander Weich, Hans Benno Leicht, Liangtao Ye, Ignazio Piseddu, Michael T. Dill, Arne Kandulski, Matthias Pinter, Ursula Ehmer, Peter Schirmacher, Jens U. Marquardt, Julia Mayerle, Enrico N. De Toni, Andreas Geier, Florian P. Reiter

**Affiliations:** ^1^ Department of Medicine II University Hospital, LMU Munich Munich Germany; ^2^ Division of Hepatology, Department of Medicine II University Hospital Würzburg Würzburg Germany; ^3^ Division of Gastroenterology, Department of Medicine II University Hospital Würzburg Würzburg Germany; ^4^ Digestive Diseases Center The Seventh Affiliated Hospital, Sun Yat‐Sen University Shenzhen China; ^5^ Department of Gastroenterology, Infectious Diseases and Intoxication Heidelberg University Hospital Heidelberg Germany; ^6^ National Center for Tumor Diseases (NCT) NCT Heidelberg, a Partnership Between DKFZ and Heidelberg University Hospital Heidelberg Germany; ^7^ German Cancer Research Center (DKFZ) Heidelberg Research Group Experimental Hepatology, Inflammation and Cancer Heidelberg Germany; ^8^ Gastroenterology, Hepatology, Endocrinology, Rheumatology and Infectious Diseases, Department of Internal Medicine I University Hospital Regensburg Regensburg Germany; ^9^ Division of Gastroenterology and Hepatology, Department of Medicine III Medical University of Vienna Vienna Austria; ^10^ Clinical Department for Internal Medicine II, Department of Clinical Medicine, TUM School of Medicine and Health, University Medical Center, Technical University of Munich Munich Germany; ^11^ Institute of Pathology University Hospital Heidelberg Germany; ^12^ Department of Medicine I University Medical Center Lübeck Germany

**Keywords:** hepatocellular carcinoma, immunotherapy, tyrosine kinase inhibition

## Abstract

**Background:**

The introduction of several new systemic therapies in recent years has significantly altered the treatment landscape for advanced hepatocellular carcinoma. However, while the approval of the combination of atezolizumab and bevacizumab as the preferred first‐line therapy over sorafenib represents progress, it has also raised uncertainties regarding optimal treatment sequencing for advanced disease.

**Aims:**

This study evaluates the sequential treatment of hepatocellular carcinoma following therapy with atezolizumab and bevacizumab, providing evidence from a prospective real‐world cohort.

**Methods:**

Data were derived from the ongoing IMMUreal cohort, which investigates immunotherapy in hepatocellular carcinoma across two tertiary centres in Bavaria. A total of 124 patients treated with atezolizumab and bevacizumab as first‐line therapy between June 2020 and December 2023 were analysed. Feasibility, treatment patterns, and outcomes of sequential therapy were assessed, with a focus on defined prognostic subgroups.

**Results:**

The median overall survival under real‐world conditions was 19.8 months. Less than half of the patients (41.2%) proceeded to second‐line therapy, and only 19.2% were eligible for third‐line treatment. This decline in treatment eligibility corresponded to a marked reduction in therapy duration and progressive deterioration in liver function, as indicated by Albumin‐Bilirubin and Child‐Pugh scores. While patients with worse baseline liver function, such as patients with Child‐Pugh B or ALBI > 1, had a significantly lower probability of transitioning to 2nd line therapy, no significant association was found between the number of treatment lines and factors such as liver cirrhosis, poor physical condition, extrahepatic disease, or macrovascular invasion.

**Conclusions:**

Sequential therapy following atezolizumab and bevacizumab is feasible only for selected patients. However, preserving liver function seems crucial to optimising multi‐line therapy and improving outcomes in advanced hepatocellular carcinoma.

## Graphical abstract

The approval of atezolizumab plus bevacizumab has improved first‐line therapy for advanced hepatocellular carcinoma, but the optimal sequencing of subsequent treatments remains uncertain. This real‐world study of 124 patients shows that only a minority advance to second‐ and third‐line therapies, with a progressive decline in liver function over sequential treatment. Preserving liver function seems therefore crucial for enabling multi‐line therapy and improving patient outcomes.

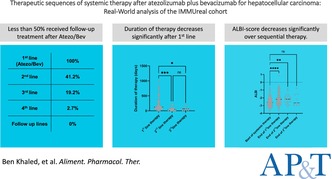

**Abbreviations**
AFP: alpha‐fetoprotein
ANOVA: analysis of variance
atezo/bev: atezolizumab and bevacizumab
CR: complete response
EASL: European Association for the Study of the Liver
EMA: European Medicines Agency
FDA: Food and Drug Administration
GROW: Guidance for Reporting Oncology Real‐World evidence
HCC: hepatocellular carcinoma
ICIs: immune checkpoint inhibitors
ipi/nivo: ipilimumab/nivolumab
MASLD: Metabolic dysfunction‐associated steatotic liver disease
mOS: median overall survival
mRECIST: modified Response Evaluation Criteria in Solid Tumours
ORR: objective response rate
PD: progressive disease
PFS: progression‐free survival
PR: partial response
QoL: quality of life
RECIST: Response Evaluation Criteria in Solid Tumours
SD: stable disease
TACE: transarterial chemoembolization
TARE: transarterial radioembolization
TKIs: tyrosine kinase inhibitors
VEGF: vascular endothelial growth factor

## 1 Introduction

Liver cancer, predominantly represented by hepatocellular carcinoma (HCC), holds a leading position among the most prevalent malignancies worldwide, with an increasing incidence [1]. In this regard, the Global Cancer Statistics 2022 report states that liver cancer is the sixth most diagnosed cancer and ranks as the third leading cause of cancer‐related deaths worldwide [1]. The majority of HCC diagnoses occur in advanced stages [2], where systemic therapy is the sole viable treatment option. Fortunately, there is a growing array of effective systemic therapies, encompassing immune checkpoint inhibitors (ICIs), vascular endothelial growth factor (VEGF) inhibitors, and tyrosine kinase inhibitors (TKIs), for the treatment of advanced HCC [3, 4, 5]. Several of these agents, either alone or in combination, have demonstrated improved outcomes compared to the longstanding standard of care, sorafenib [4, 6, 7].

However, for almost a decade, sorafenib was the only therapy with proven efficacy for advanced HCC. This changed in 2017 with the approval of regorafenib, a TKI that demonstrated efficacy in 2nd line treatment for patients who tolerated sorafenib in 1st line [8]. Since then, the therapeutic armamentarium for HCC has expanded significantly, with further TKI‐based regimens [9, 10] and the emergence of the first immunotherapy combination, atezolizumab and bevacizumab (atezo/bev). This combination demonstrated for the first time superiority over the long‐standing standard sorafenib in the 1st line [11] across key endpoints, including median overall survival (mOS), progression‐free survival (PFS), and objective response rate (ORR) [11]. With its approval by the European Medicines Agency (EMA) on October 27th, 2020, atezo/bev became the preferred first‐line therapy for HCC in Europe. This paradigm shift in HCC treatment has been further amplified by various other ICI‐based regimens. Durvalumab with or without tremelimumab [12] has gained Food and Drug Administration (FDA) and EMA approval in the 1st line, while Pembrolizumab [13] and ipilimumab/nivolumab (ipi/nivo) [14] are FDA‐approved for the 2nd line after sorafenib (June 2024). However, 1st line approval of ipi/nivo is anticipated following the promising results of the CheckMate 9DW trial [15]. The therapeutic spectrum for HCC has been broadened by the availability of newer TKIs such as lenvatinib in 1st line or cabozantinib in 2nd or 3rd line after sorafenib [9, 10]. Additionally, the anti‐VEGF inhibitor ramucirumab has been approved for patients with alpha‐fetoprotein (AFP) ≥ 400 ng/mL in 2nd line following sorafenib [16].

The rapid expansion of systemic therapies coupled with the replacement of sorafenib by atezo/bev as the 1st line standard [17, 18] has introduced new complexities in the selection of treatment options. Most agents have exclusively been investigated in comparison to or following sorafenib, which no longer represents the standard 1st line therapy for HCC [6, 17, 18]. With the new variety of therapeutic options and the absence of a defined standard or prospective data post‐atezo/bev, real‐world data on treatment sequences are essential. Understanding the feasibility and outcomes of sequential therapy is crucial in a malignancy such as HCC, which typically impacts liver function with each progression [19].

The IMbrave 150 trial reported that only 20.5% of patients received 2nd line therapy after atezo/bev, though reasons for this low rate were not reported, to our knowledge [11]. As a result, the feasible number of treatment lines and outcomes after atezo/bev remains uncertain. Investigating the feasibility of multiple therapy lines is critical to inform future strategies to optimise HCC management. Tumour progression in HCC frequently occurs within the liver—a vital organ that is often compromised by the underlying chronic liver disease. Early progression and hepatic decompensation have been shown to correlate with reduced OS following atezo/bev treatment [20, 21]. If only a small proportion of patients can proceed to 2nd or 3rd line therapies, a strategic shift toward optimising, intensifying, or more precisely selecting 1st line therapeutic approaches should become a focus.

Considering these factors, our study delves into the applied therapeutic sequences within the prospective IMMUreal cohort. This cohort provides data from two tertiary centres in Bavaria, Germany, where reimbursement for 2nd and follow‐up line treatments is generally not constrained by the healthcare provider. This analysis aims to reflect the feasibility of sequential therapy for HCC in real‐world scenarios following atezo/bev.

## 2 Patients and Methods

### 2.1 Patient Population

Patient data were sourced from the IMMUreal cohort, a prospective study investigating the application of ICI‐based therapy in patients with liver cancer. The cohort included data from two tertiary study sites, the University Hospital Würzburg and University Hospital LMU Munich. For this study, only patients were included who received atezo/bev in 1st line. The data cutoff was performed in May 2024. HCC diagnosis relied on histopathological findings or typical diagnostic imaging, adhering to the criteria outlined by the European Association for the Study of the Liver (EASL) [22, 23]. This study received approval from local authorities (Ethikkommission an der Julius‐Maximilians‐Universität Würzburg, 156/21‐me) and adhered to the principles of the Declaration of Helsinki. All patients provided informed consent. We employed the STROBE cohort checklist and followed the ESMO Guidance for Reporting Oncology Real‐World evidence (GROW) [24, 25]. Data was analysed retrospectively.

### 2.2 Treatments

Patients received the following treatment regimens: Atezolizumab combined with bevacizumab, involving intravenous administration of atezolizumab at a dosage of 1200 mg and bevacizumab at 15 mg per kg of body weight every 3 weeks [11]. Lenvatinib was prescribed at 12 mg orally once daily for patients weighing ≥ 60 kg and at 8 mg once daily for patients weighing < 60 kg [9]. Regorafenib was introduced if sorafenib was tolerated in previous lines, with a dosage of 160 mg for 3 weeks followed by a 1‐week pause [8]. Cabozantinib was prescribed at 60 mg per day [10]. Pembrolizumab was administered at 200 mg every 3 weeks [13]. The combination of ipilimumab/nivolumab was administered based on arm A “Ipi^high^” from the Checkmate‐040 study, comprising nivolumab at 1 mg/kg plus ipilimumab at 3 mg/kg every 3 weeks for 4 doses, followed by nivolumab at 240 mg intravenously every 2 weeks [14]. Sorafenib was administered at 800 mg per day [6]. Durvalumab and Tremelimumab were administered according to the STRIDE protocol with a single dose of Tremelimumab at 300 mg and repeated Durvalumab at 1500 mg Q4W [12].

In cases of intolerance, individual dose reductions of the specified therapy protocols were applied according to the EMA approved conditions of use.

Throughout treatment, patients were systematically monitored through clinical, laboratory, and imaging assessments following the standard of care in adherence to the German HCC guidelines [18]. During visits, vital signs were measured, and laboratory tests included a complete blood count, serum chemistry, parameters of liver function, and AFP. An adverse events assessment was performed at each visit. Tumour response was evaluated every 8‐ to 12‐week using computed tomography and/or magnetic resonance imaging. Follow‐up was conducted for all patients.

### 2.3 Study Objectives

**FIGURE 1:**
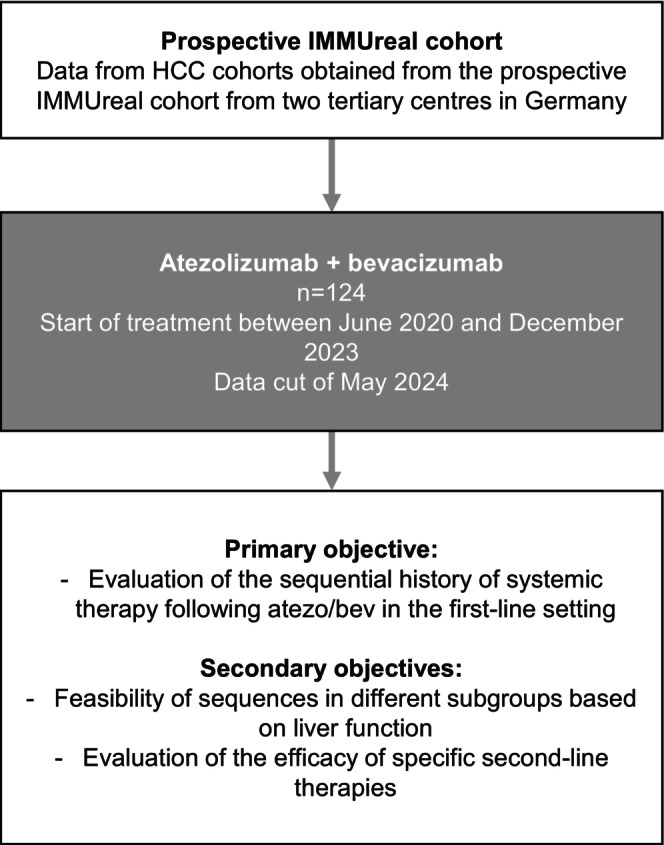
Study flow chart.

The primary objective of this study was to examine the treatment sequences applied in patients who received atezo/bev as a 1st line therapy in a real‐world setting. Secondary objectives included analysing the liver function under sequence therapy and evaluating the efficacy of specific 2nd line therapies (Figure 1). The mOS was defined as the time from treatment initiation to death from any cause. Patients without an OS event or those lost to follow‐up were censored on their last contact day. Treatment response was assessed through routine computed tomography or magnetic resonance imaging until death or the end of treatment. Radiological response was categorised as complete or partial response (CR/PR) or stable or progressive disease (SD/PD), either determined by the local investigator and radiologist and/or in accordance with Response Evaluation Criteria in Solid Tumours (RECIST) version 1.1 or modified RECIST (mRECIST).

### 2.4 Statistical Analysis and Illustration

Statistical analyses were conducted using GraphPad Prism 9 (GraphPad Software, San Diego, CA, USA) using analysis of variance (ANOVA) or the Mann–Whitney U test as indicated. Descriptive statistics were employed to summarise baseline characteristics. Regarding HCC aetiology in patients with mixed liver diseases, we categorised patients according to the aetiology with the highest oncogenic potential using this hierarchy: Hepatitis C (untreated/treated), alcohol, Hepatitis B, Metabolic dysfunction‐associated steatotic liver disease (MASLD), other causes of liver cirrhosis, non‐cirrhotic liver. Categorical variables were presented as numbers and percentages. For the calculation of mOS, Kaplan–Meier analysis was performed, with censoring applied for patients alive at last follow‐up, lost to follow‐up, or for patients undergoing liver transplantation. Logistic regression was used to assess factors associated with transitioning to 2nd line therapy. Patients discontinuing 1st therapy without progressing to 2nd line therapy, such as patients dying on 1st therapy, patients receiving BSC, or patients lost to follow‐up, were censored. Patients with ongoing 1st line therapy or patients with local treatment modalities following 1st line therapy (transplant, resection, locoregional therapies) were excluded. Odds ratios (ORs) with 95% confidence intervals (CIs) were calculated. Statistical significance was determined at *p*‐values below 0.05. The therapeutic pathway was illustrated using a Sankey diagram. This was achieved by an open source application (https://sankeymatic.com).

### 2.5 Study Design

The source data were obtained from the prospective IMMUreal cohorts, comprising all patients who received atezo/bev as a 1st line treatment at the participating centers (University Hospital Würzburg and University Hospital LMU Munich).

### 2.6 Exclusion Criteria

The therapy decisions were made at the physician's discretion. With the primary objective of capturing a genuine real‐world context, no exclusions were made among HCC patients, except for those who declined informed consent or did not undergo atezo/bev as a 1st line treatment. Patients with combined HCC/CCA tumours or fibrolamellar HCCs were not considered in this study.

### 2.7 Data Source and Study Data Management

The data utilised in this study were extracted from medical records and patients' reports during the recruitment phase for observational prospective patient cohorts at each participating center. Each center contributed source data in an anonymized format through a predefined form, and the merger of all data for subsequent analysis was facilitated by VZ. Notably, the analysis revealed no instances of duplicated cases, a phenomenon likely influenced by the diverse geographical locations. The compilation of datasets occurred in May 2024. Following this, FPR and NBK verified data completeness, and comprehensive quality controls and validation procedures were subsequently executed.

## 3 Results

### 3.1 Baseline Characteristics

Data from 124 patients who received atezo/bev as 1st line treatment between June 2020 and December 2023 were analysed in this study (Figure 1). Baseline characteristics are detailed in Table 1. The majority of patients, 93 (75%), had underlying liver cirrhosis. Most were classified as Child‐Pugh A (76, 81.7%), while 16 patients (17.2%) had Child‐Pugh B. Only one patient was categorised as Child‐Pugh C (1.1%). A large proportion of patients had preserved performance status, with 92 (74.2%) having an ECOG score of 0, and 28 (22.6%) presenting with an ECOG score of 1. Only four patients (3.2%) received therapy with an ECOG score ≥ 2. Fifty‐three patients had undergone prior local therapy (42.7%), encompassing a total of 64 procedures, primarily resection, microwave or radiofrequency ablation, and transarterial chemoembolization (TACE). The underlying aetiology was viral in 30 patients (24.2%), non‐viral in 83 patients (66.9%) and unknown in 11 patients (8.9%). Among the 30 patients with viral aetiology, 25 tested positive for HCV antibodies. Of these, 13 had viremia at the start of atezo/bev treatment. Five patients were positive for HBV only, while another five had a combined HBV/HCV aetiology. In total, 14 patients in this study had more than one aetiology for HCC. Extrahepatic spread and macrovascular invasion were observed in 46 (37.1%) and 32 (25.8%) patients, respectively.

**TABLE 1:** Baseline characteristics at start of atezo/bev.

Patient characteristics	Atezo/Bev (*n* = 124)
Age at start of atezo/bev, median (range)	68 (38–87)
Sex, female, *n* (%)	23 (18.5)
Liver cirrhosis, *n* (%)	93 (75)
Child‐Pugh A	76 (81.7)
Child‐Pugh B	16 (17.2)
Child‐Pugh C	1 (1.1)
*BCLC stage, n (%)*	
BCLC A	6 (4.8)
BCLC B	41 (33.1)
BCLC C	76 (61.3)
BCLC D	1 (0.8)
*ECOG performance status, n (%)*	
ECOG 0	92 (74.2)
ECOG 1	28 (22.6)
ECOG 2	2 (1.6)
ECOG 3	2 (1.6)
*Prior non systemic therapy*	
*n* of patients/*n* of procedures	53/64
Resection (% of all procedures)	20 (31.3)
MWA/RFA (% of all procedures)	9 (14.1)
TACE (% of all procedures)	27 (42.2)
TARE/SBRT/other (% of all procedures)	8 (12.5)
Transplantation (% of all procedures)	0 (0)
*Aetiology of underlying liver disease, n (%)*	
HBV/HCV	30 (24.2)
Non‐viral	83 (66.9)
Unknown	11 (8.9)
*Extrahepatic spread, n (%)*	
No extrahepatic spread	78 (62.9)
Extrahepatic spread present	46 (37.1)
*Macrovascular invasion, n (%)*	
No macrovascular invasion	91 (73.4)
Macrovascular invasion present	32 (25.8)
Unknown	1 (0.8)
*Alpha‐fetoprotein levels (AFP levels), n (%)*	
AFP > 400 ng/mL	32 (25.8)
AFP ≤ 400 ng/mL	84 (67.7)
No AFP levels available	8 (6.5)
*ALBI‐score, n (%)*	
ALBI score 1	61 (49.2)
ALBI score 2	57 (46.0)
ALBI score 3	6 (4.8)

Abbreviations: AFP, Alpha‐fetoprotein; ALBI‐Score, Albumin‐Bilirubin‐Score; BCLC, Barcelona Clinic Liver Cancer; ECOG, Eastern Cooperative Oncology Group; HBV, hepatitis B virus; HCV, hepatitis C virus; MWA, microwave ablation; n, number; RFA, radiofrequency ablation; SBRT, Stereotactic Body Radiation Therapy; TACE, transarterial chemoembolization; TARE, transarterial radioembolization.

### 3.2 Systemic Treatment Sequencing

**FIGURE 2:**
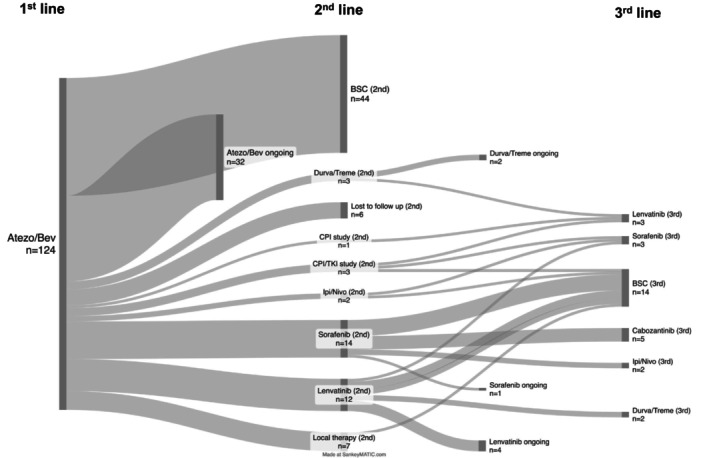
Therapeutic pathways of sequential systemic therapy in advanced HCC following atezo/bev. The Sankey diagram illustrates the therapeutic sequences subsequent to atezo/bev in first‐line therapy (*n* = 124). (This diagram was created using an open‐source program, available at https://sankeymatic.com).

At the time of data cutoff, 65 participants (52.4% of the whole population) had died (Table 2). The mOS from atezo/bev initiation was 19.8 months (601 days; *n* = 124; Figure S1). After excluding 19 patients with missing response assessment data, the best ORR was 31.5% (Table 3), comprising CR in 3 patients and PR in 36 patients. SD was observed in 34 patients. Disease control was achieved in 73 patients, corresponding to a DCR of 58.9%. At cutoff, 32 patients (25.8%) were still undergoing 1st line treatment with atezo/bev (Figure 2 and Table 2). Among the remaining 92 patients, 7 (5.6%) received additional local therapies. During 1st line therapy, 22 patients (23.9%) died (Table 2), and 45 (48.9%) discontinued treatment due to PD. Other reasons for therapy discontinuation included toxicity (*n* = 8, 8.7%), patient preference (*n* = 4, 4.3%), or other causes (*n* = 13, 14.1%, for specifications, see Table S1 and Table 2). At the data cutoff, 35 patients (28.2%) received 2nd line systemic therapy. Considering these patients and excluding those continuing with atezo/bev (*n* = 32) or those shifted to local therapy (*n* = 7), a total of 41.2% received 2nd line therapy following atezo/bev. Additionally, at the data cutoff, 15 patients (12.1%) received 3rd line, and 2 patients (1.6%) 4th line systemic therapy. Excluding patients still on 2nd (*n* = 7) or 3rd line (*n* = 3) systemic therapy, 19.2% and 2.7% of all patients received 3rd or 4th line systemic therapy, respectively. No patient received therapy beyond the 4th line at the time of data cutoff. Reasons for therapy discontinuation up to the 3rd line are detailed in Table 2. Forty‐four patients (35.5%) received only atezo/bev (Figure 2). Of these 44 patients, some either died during first‐line therapy or received best supportive care (BSC) after atezo/bev. This group is summarised as the BSC group in Figure 2. The mOS for this group from the start of atezo/bev in the first line was 13.6 months (413 days; *n* = 44; not shown).

**TABLE 2:** Overview of therapeutic lines.

Overview of therapeutic sequences, survival status and reasons for follow up therapy after first line	Atezo/Bev (*n* = 124)
*Therapeutic line, n (%)* First line (at least)/therapy ongoing/change to local therapy Second line (at least)/therapy ongoing Third line (at least)/therapy ongoing Follow up lines (at least)/therapy ongoing/local therapy	124 (100)/32 (25.8)/7 (5.6) 35 (28.2)/7 (5.6) 15 (12.1)/3 (2.4) 2 (1.6)/0 (0)/1 (0.8)
*Survival status, n (%)* Alive Deceased Unknown	53 (42.7) 65 (52.4) 6 (4.8)
*Reasons for disruption of first line therapy, n (%)* Progressive disease Intolerance to medication Patient deceased Patient's wish Other reasons	45 (48.9) 8 (8.7) 22 (23.9) 4 (4.3) 13 (14.1)
*Reasons for disruption of second line therapy, n (%)* Progressive disease Intolerance to medication Patient deceased Patient's wish Other reasons	12 (42.9) 9 (32.1) 5 (17.9) 1 (3.6) 1 (3.6)
*Reasons for disruption of third line therapy, n (%)* Progressive disease Intolerance to medication Patient deceased Patient's wish Other reasons	4 (33.3) 2 (16.7) 4 (33.3) 2 (16.7) 0 (0.0)

Abbreviation: N, number.

**TABLE 3:** Overview of therapeutic sequences, survival status and reasons for discontinuation.

Overview of duration of therapy and best response per line	*n* = 124
*Duration of therapy per line in days (standard deviation)* First line Second line Third line	236 (±226) 64.7 (±63.7) 71.3 (±55.4)
*Best response first line therapy with Atezo/Bev, n (%)* CR PR SD PD Mixed response Missing data ORR (% of all available response assessments) DCR (% of all available response assessments) mOS months (days)	3 (2.4) 36 (29.0) 34 (27.4) 31 (25.0) 1 (0.8) 19 (15.3) 39 (31.5) 73 (58.9) 19.8 (601)
*Best response second line therapy, n (%)* CR PR SD PD Missing data ORR DCR mOS months (days)	1 (2.9) 2 (5.7) 6 (17.1) 8 (22.9) 18 (51.4) 3 (8.6) 9 (25.7) 5.0 (152)
*Best response third line therapy, n (%)* CR PR SD PD Missing data ORR DCR mOS months (days)	0 (0.0) 1 (6.7) 2 (13.3) 6 (40.0) 6 (40.0) 1 (6.7) 3 (20) 4.1 (125)

Abbreviations: CR, complete remission; *n*, number; PD, progressive disease; PR, partial remission; SD, stable disease.

The mOS from the initiation of 2nd line therapy was 5.0 months (152 days; *n* = 34; Supp. Figure 1). In the 2nd line, the ORR was 8.6%, with one patient achieving CR and two patients achieving PR (Table 3). Six patients experienced SD as the best response, resulting in a DCR of 25.7% (Table 3). Most patients received TKI‐based therapy in the 2nd line (Figure 2). Sorafenib was the most commonly administered TKI, with 14 patients (40%), followed by lenvatinib (12 patients, 34.3%). Nine patients (25.7%) received an immune checkpoint inhibitor‐based regimen in the 2nd line after atezo/bev, with 4 of these enrolled in clinical trials (Figure 2).

3rd line systemic therapy was administered to only a small number of patients (15, 12.1%), while 14 patients received BSC after 2nd line systemic therapy. The mOS from the initiation of 3rd line therapy was 4.1 months (125 days; Supp. Figure 1). The ORR in the 3rd line was 6.7%, with 1 patient achieving PR (Table 3). Two patients reached SD as the best response, resulting in a DCR of 20% (Table 3).

Missing response data increased with the progression of therapeutic lines. 19 patients (15.3%) had missing data in the 1st line, 18 (51.4%) in the 2nd line, and 6 (40.0%) in the 3rd line.

**FIGURE 3:**
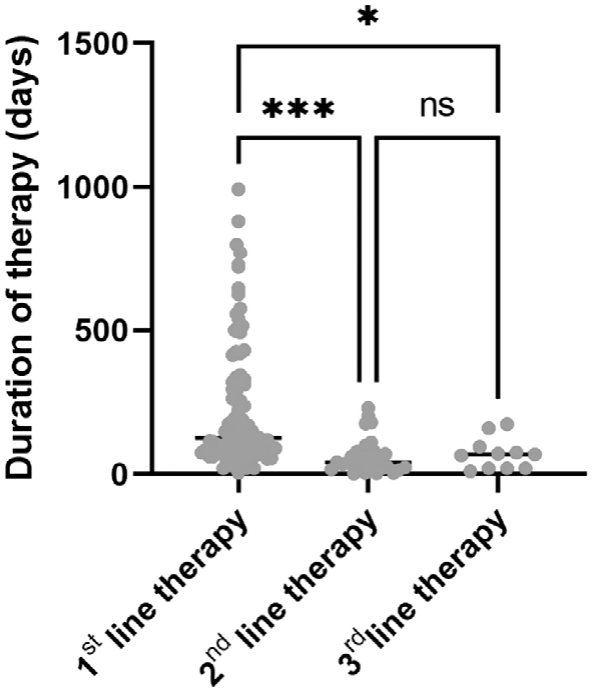
Duration of Therapy. The duration of each therapeutic line up to the 3rd line is illustrated (*n* = 11 to 87; ***p < 0.001; *p < 0.05; ANOVA).

The median duration of therapy at data cut‐off was significantly reduced after the 1st line. The median duration of treatment was 236 ± 226 days for the 1st line, 64.7 ± 63.7 days for the 2nd line, and 71.3 ± 55.4 days for the 3rd line (Table 3 and Figure 3).

### 3.3 Patterns of Local Therapy in Atezo/Bev‐Treated Patients

Since the use of local therapy after the initiation of atezo/bev represents an exceptional scenario, we conducted a detailed analysis of this subgroup. In the subgroup (*n* = 7) that received local therapy after the initiation of atezo/bev, one patient achieved a PR under atezo/bev, three patients had SD, one patient showed a mixed response, and two patients experienced PD. As local therapy, a liver transplant was performed in three patients, of whom one achieved PR and two had SD under atezo/bev. Additionally, one patient with SD under atezo/bev underwent tumour resection. One patient with PD under atezo/bev was treated with TACE targeting only the progressive lesions. Another patient with PD received transarterial radioembolization (TARE) for all lesions. The patient who showed a mixed response was also treated with TARE, targeting all lesions. None of the seven patients continued atezo/bev therapy after local therapy.

### 3.4 Determinants of Treatment Choice and Outcomes in the 2nd Line Setting

Given that lenvatinib and sorafenib were the preferred choices for 2nd line therapy, we evaluated the factors influencing treatment selection. Among the 7 patients treated with lenvatinib, the primary reason for therapy choice was the potential benefit in terms of mOS or mPFS, while in 3 patients, a combination of benefit in mOS or mPFS and likelihood of response influenced the decision. One patient had a preference to receive lenvatinib in 2nd line. In the sorafenib group, in 5 patients the primary reason for choice of therapy was benefit in terms of mOS or mPFS, while in 6 patients, a combination of mOS/mPFS benefit and likelihood of response guided the decision. In one patient, the reason was reduced quality of life (QoL), and in another patient, reduced ECOG.

Taken together, there was no clear reason for the choice of therapy identifiable, while in two patients, concerns with respect to QoL or reduced ECOG led to the use of sorafenib, which was not evident in patients treated with lenvatinib.

We identified 5 patients who received dual ICI therapy following atezo/bev in the 2nd line outside of clinical studies. Given the rarity of initiating ICI therapy immediately after a previous ICI regimen, we conducted a detailed analysis of these cases. Three of these patients received durvalumab plus tremelimumab after atezo/bev, with two patients remaining on this regimen at the time of data cutoff. The remaining 2 patients received ipi/nivo in the 2nd line after atezo/bev. Of these five patients, 2 had PR as the best response under atezo/bev, while 1 achieved SD, and 2 had PD with atezo/bev. The choice of 2nd line ICI therapy was driven by relevant comorbidities precluding TKI therapy in three patients and the adverse effect profile in 1 patient. Likelihood of response influenced the choice in 1 patient. The best response with 2nd line ICI was a CR in 1 patient, SD in 2 patients, and PD in 1 patient. Response assessment was not available for one patient at the time of data cutoff.

Since sorafenib and lenvatinib were the preferred therapies in the 2nd line, we performed an efficacy analysis between these two regimens. Data from 14 sorafenib‐treated patients and 11 lenvatinib‐treated subjects were included. The mOS was 4.8 months for sorafenib and 7.7 months for lenvatinib. No statistically significant differences were found between the two therapies (Figure S2).

### 3.5 Longitudinal Evaluation of Liver Function Throughout Treatment Sequencing

**FIGURE 4:**
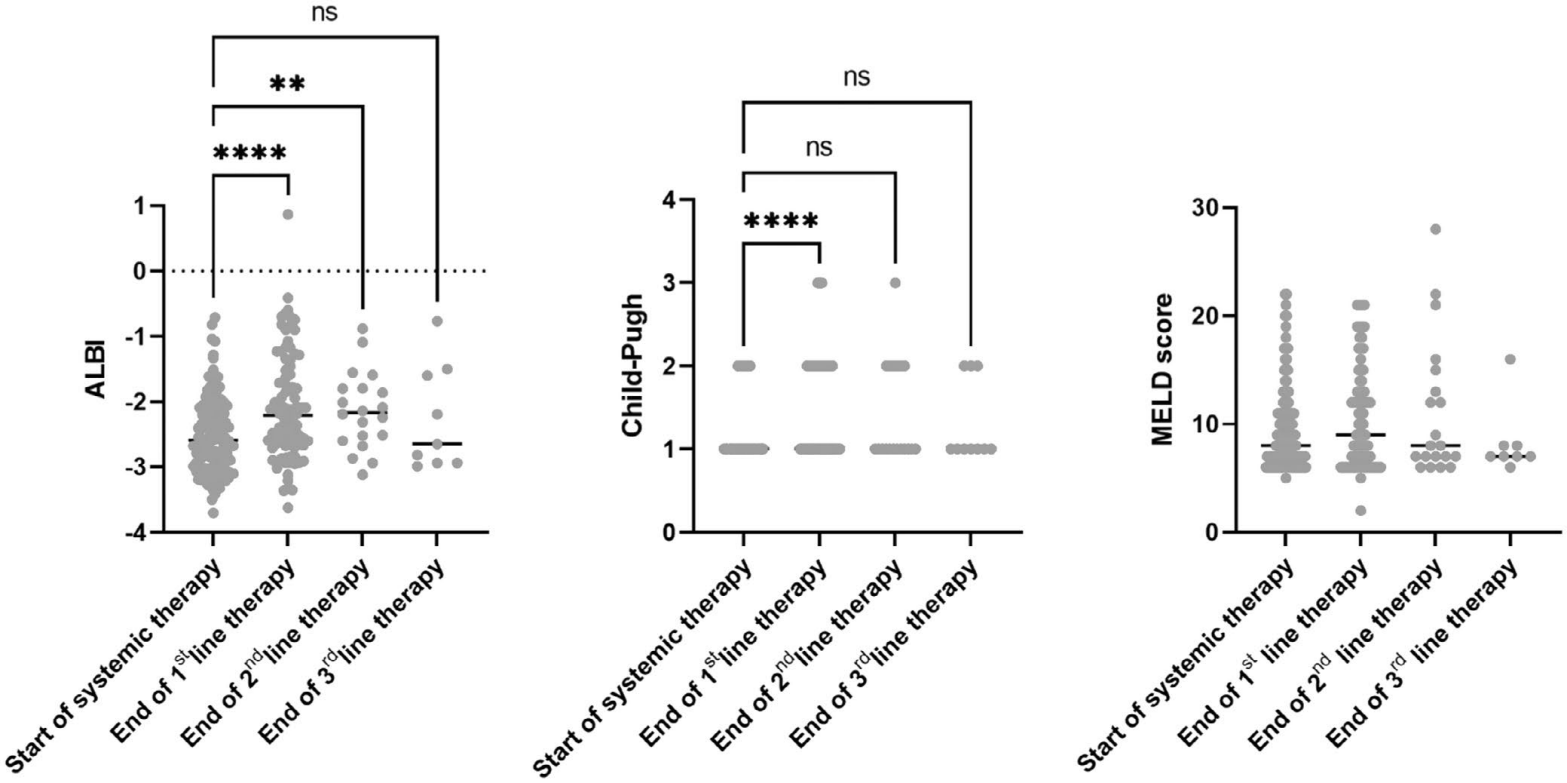
Development of liver function under sequential systemic therapy for HCC. The figure illustrates the development of liver function from the start of systemic therapy to the end of each therapeutic line. The Child‐Pugh score is indicated as Child A = 1, Child B = 2, and Child C = 3. For this analysis, patients who had ongoing therapy or received best supportive care were excluded (*n* = 10 to 108; **** *p* < 0.0001; **p < 0.01; ANOVA).

Deterioration of liver function could represent a relevant limiting factor for applying sequential therapy for HCC. Therefore, we aimed to analyse the course of liver function over time using the MELD, Child‐Pugh, and ALBI scores after the 1st, 2nd, and 3rd lines of therapy. The mean ALBI score was −2.52 ± 0.58 at the start of therapy and decreased significantly to −2.08 ± 0.82 at the end of the 1st line, −2.14 ± 0.59 at the end of the 2nd line, and − 2.27 ± 0.81 at the end of the 3rd line. The Child‐Pugh score deteriorated significantly, with more patients reaching a Child‐Pugh B or C by the end of the 1st line of therapy. In contrast, the MELD score did not show significant changes throughout the course of therapy. These findings indicate a notable decline in liver function, as reflected by the ALBI and Child‐Pugh scores, over the course of therapy. Patients receiving best supportive care or those with ongoing treatments were excluded from this analysis (Figure 4).

### 3.6 Feasibility of Sequential Therapy for HCC in Certain Subgroups

**FIGURE 5:**
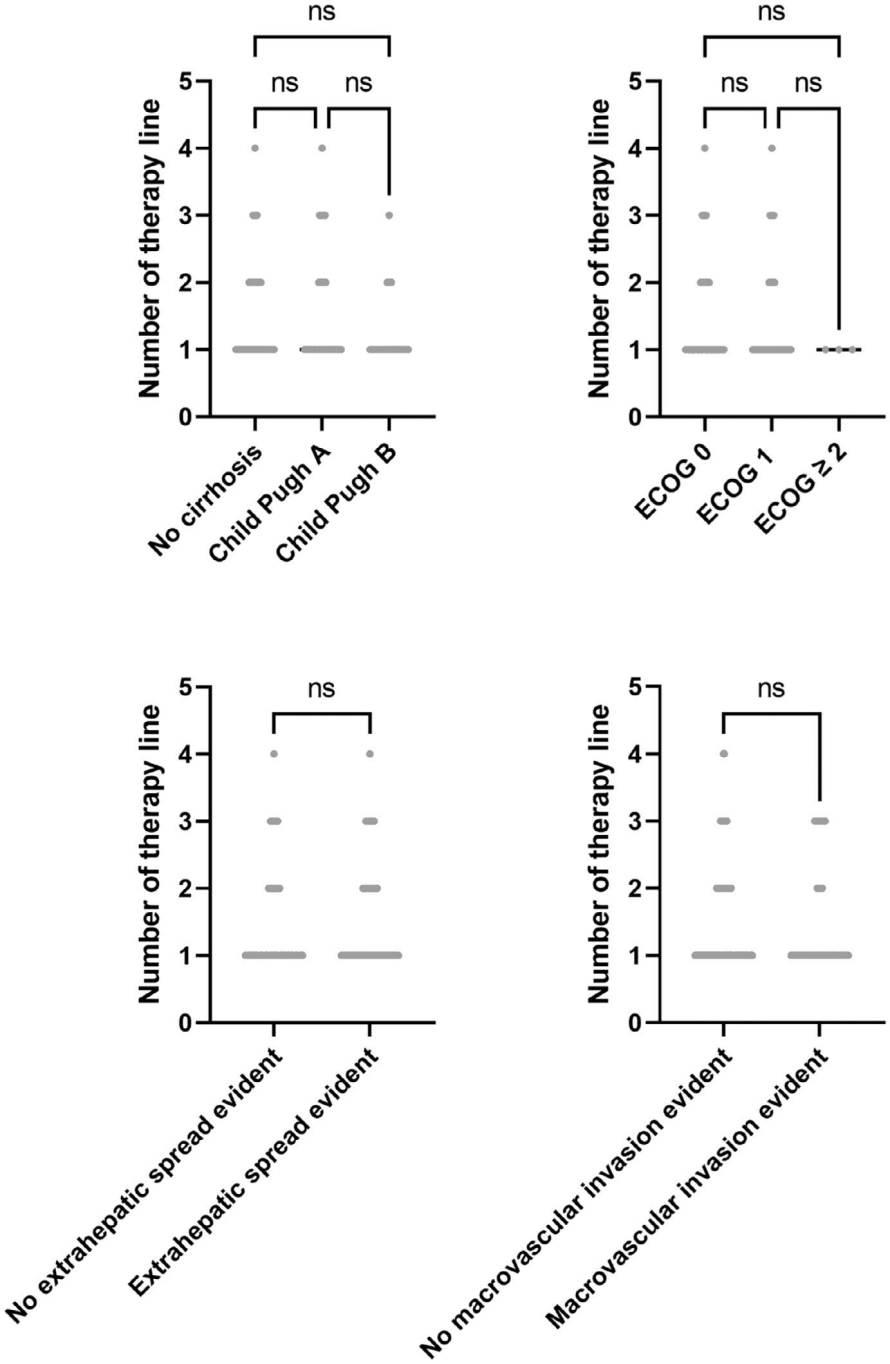
Number of therapeutic lines in different prognostic subgroups. The figure illustrates the number of applied therapeutic lines in patients with liver cirrhosis, reduced performance status, evidence of extrahepatic spread, or presence of macrovascular invasion (*n* = 3 to 93; ANOVA or Mann–Whitney *U* test).

In a further analysis, we investigated the feasibility of sequential therapies in certain subgroups. Prognostically relevant factors such as cirrhosis, poor performance status, extrahepatic spread, or macrovascular invasion were examined. We did not find a statistically significant influence of these parameters on the number of administered therapeutic lines (Figure 5). To analyse factors influencing the likelihood of transitioning to 2nd line therapy, we performed a logistic regression analysis. Patients with worse baseline liver function, such as patients with Child Pugh B or ALBI > 1, had a significantly lower probability of transitioning to 2nd line therapy (Child Pugh B: *p* = 0.0143, ALBI > 1: *p* = 0.0097, see Table S2). Other factors did not significantly influence the likelihood of transitioning to 2nd line, including baseline characteristics such as age, sex, ECOG, BCLC stage or presence of extrahepatic spread or macrovascular invasion.

## 4 Discussion

In this study, we present findings on the feasibility and outcomes of sequential therapy for HCC based on two prospective cohorts (IMMUreal cohort). With recent advances in systemic therapy for HCC, including the replacement of sorafenib as a 1st line standard and the approval of several new therapeutic options [3, 4], the optimal treatment strategy after atezo/bev remains unclear. Prospective randomised trials reporting on therapies following atezo/bev are scarce [26]. The results of the IMbrave251 trial (NCT04770896), investigating the combination of atezo plus sorafenib or lenvatinib versus sorafenib or lenvatinib alone as a 2nd line approach after atezo/bev, are eagerly awaited. Real‐world cohorts can provide robust and meaningful evidence on the sequential therapy of HCC until data from high‐quality randomised trials become available. However, results from randomised clinical trials may have limitations when applied to real‐world populations, as these trials often employ strict selection criteria that may not fully reflect scenarios from clinical practice. The data presented are particularly valuable because they reflect real‐world conditions, including patients who did not meet the eligibility criteria of the IMbrave150 trial.

Regarding the feasibility of sequential therapy after atezo/bev, we observed that after excluding patients still receiving atezo/bev at the time of data cutoff (*n* = 32), 41.2% of patients received follow‐up systemic treatment. This transition rate is lower than reported in recently published 2nd line cohorts. Persano et al. found that around 50% of patients in a mixed Western/Eastern cohort transitioned to 2nd line [27], while a recent Asian cohort showed a transition rate of 70% [28]. The differences in transition rates could partly be attributed to variations in baseline patient characteristics. Both the Persano et al. and Chon et al. cohorts contained a high proportion of patients with viral HCC and preserved liver function (Child‐Pugh A) [27, 28]. In contrast, our study focused on a Western cohort with predominantly non‐viral aetiology and a higher percentage of patients with poorer liver function (Child‐Pugh B). Our logistic regression analysis confirmed that worse baseline liver function, defined by Child‐Pugh B or ALBI > 1, was significantly predictive of a lower likelihood of transitioning to second‐line therapy. Despite the lower transition rate compared to other real‐world cohorts, our finding of 41.2% is notably higher than the 20.5% reported in the IMbrave150 trial [11]. This discrepancy may reflect restricted reimbursement policies in certain regions where IMbrave150 was conducted. Additionally, the broader routine adoption of immunotherapy for HCC in clinical practice, a trend previously noted with sorafenib [29], could contribute to higher transition rates in real‐world studies.

Regarding the efficacy of 2nd line therapy, the mOS of 5.0 months observed in our study is markedly lower than the 7.5 to 16.6 months reported in Asian cohorts and the 14.2 to 17.0 months reported in the global cohort of Persano et al. [27, 28, 30, 31, 32]. ORR was 8.6% in our study, compared to 6.1% to 25% in Asian cohorts [30, 31, 32]. Persano et al. did not report response rates. The poorer efficacy data in our study could be related to the cohort's poorer baseline liver function. The poorer efficacy outcomes in our cohort are likely attributable to the worse baseline liver function of our patients, underscoring the significant impact of liver function on treatment outcomes in sequential therapy settings.

In our study, nearly 48% (*n* = 44) of patients were deemed ineligible for 2nd line therapy after excluding those who were still receiving atezo/bev at data cutoff. This observation underscores the aggressive nature of HCC progression, which often manifests in deterioration of liver function [19] or complications driven by portal hypertension [33]. We observed a significant worsening of liver function over time, as assessed by ALBI and Child‐Pugh scores. A limitation of this study is the inability to clearly differentiate whether liver function deterioration was due to tumour progression, toxicity, or the natural course of liver disease. Nevertheless, the decline in liver function during sequential therapy is undeniable and must be addressed in future trials. From a safety perspective, intensifying first‐line therapy to prevent HCC progression and worsening of liver function may improve patient outcomes, as assessed by ongoing prospective trials like IMbrave152 (NCT05904886) [28] investigating atezo/bev + tiragolumab, and the Montblanc trial (NCT05844046) [29], exploring durvalumab/tremelimumab + bevacizumab. These triplet therapies represent promising approaches for future HCC systemic therapies.

German guidelines recommend sorafenib as systemic therapy for patients eligible for treatment after progression or intolerance to atezo/bev [34]. However, there are also expert opinions advocating for the use of lenvatinib following atezo/bev [35, 36]. Evidence to support the usage of TKI after atezo/bev is limited, and to the best of our knowledge, no prospective randomised trials are available. In our study, we observed that a notable proportion of patients received lenvatinib (*n* = 12) as the second‐line option. One potential reason might be the significantly better PFS (7.4 vs. 3.7 months) and time to progression (8.9 vs. 3.7 months) observed with lenvatinib compared to sorafenib in the 1st line setting [9]. Additionally, the higher ORR observed with lenvatinib compared to sorafenib (40.6% vs. 12.4% according to mRECIST) [9] may also contribute to the preference for lenvatinib. Another reason for selecting lenvatinib as the follow‐up therapy of choice could be the clinically meaningful mOS of 19 months reported in the lenvatinib control arm (lenvatinib + placebo) of the LEAP‐002 trial [37]. In our study, we identified a numerically higher mOS for Lenvatinib compared to sorafenib, which did not reach statistical significance (Figure S2). However, due to the low sample size, the interpretation of these results is limited.

As response rates improve with modern systemic therapies, downstaging of patients with advanced HCC may become more common. In this regard, we observed a subset of 7 patients who were treated with local therapies during the course of systemic treatment. This development may represent a paradigm shift in HCC therapy. Future studies are needed to investigate the course of this particular subgroup, which will likely be encountered more frequently as response rates continue to increase.

There are certain limitations to the current analysis. First, the study was conducted in tertiary centres, which may limit the broader applicability of the results presented. Second, the sample size for certain analyses was small, which limits the statistical power of the analyses. The study compensates for these limitations by providing an in‐depth characterisation of treatment sequencing in a prospectively followed cohort of HCC patients.

A further limitation is that response assessments according to RECIST and mRECIST were not available for every patient, which is often the case in real‐world settings, where logistical constraints in clinical practice do not always allow all methods to be applied to every patient. However, in this study, we used investigator‐based assessments, which can be considered homogeneous, as both centres worked closely together, and the leading investigator was trained at the other centre, providing a consistent approach to assessment.

While PD is the essential parameter for making decisions about further systemic treatment sequences, regardless of whether RECIST or mRECIST is used, the granularity in terms of PR or CR differs between the two methods. Both RECIST and mRECIST identify PD almost similarly, but they may yield different results for PR and CR. However, as the main goal of this paper is to provide a comprehensive understanding of therapeutic sequences in systemic HCC treatment, we believe we have appropriately addressed this question, despite a limitation in the granularity of PR and CR assessments.

In summary, we report that approximately 40% of patients were able to receive follow‐up therapy after 1st line treatment with atezo/bev. Deterioration of liver function following 1st line therapy may significantly limit the feasibility of further treatments. Our findings suggest that new strategies are needed to improve outcomes in sequential systemic treatments for HCC, either by intensifying first‐line therapies or by refining patient selection using biomarkers to predict therapeutic efficacy. Striking the right balance between treatment intensity and liver function preservation is crucial for managing advanced HCC; prospective studies are necessary to address these challenges.

## Author Contributions

**Najib Ben Khaled:** conceptualization, investigation, writing – original draft, methodology, validation. **Valentina Zarka:** methodology, validation, visualization, software, data curation. **Bernard Hobeika:** methodology, validation, visualization, software, data curation. **Julia Schneider:** methodology, validation, visualization, software, data curation. **Monika Rau:** writing – review and editing. **Alexander Weich:** writing – review and editing. **Hans Benno Leicht:** writing – review and editing. **Liangtao Ye:** writing – review and editing. **Ignazio Piseddu:** writing – review and editing. **Michael T. Dill:** writing – review and editing. **Arne Kandulski:** writing – review and editing. **Matthias Pinter:** writing – review and editing. **Ursula Ehmer:** writing – review and editing. **Peter Schirmacher:** writing – review and editing. **Jens U. Marquardt:** writing – review and editing. **Julia Mayerle:** writing – review and editing. **Enrico N. De Toni:** writing – review and editing. **Andreas Geier:** writing – review and editing. **Florian P. Reiter:** conceptualization, investigation, funding acquisition, writing – original draft, methodology, validation, visualization, writing – review and editing, software, formal analysis, project administration, data curation, supervision, resources.

## Disclosure

**Approval Statement:** All authors approved the final version of the manuscript.

## Ethics Statement

This study received approval from local authorities (Ethikkommission an der Julius‐Maximilians‐Universität Würzburg, 156/21‐me) and adhered to the principles of the Declaration of Helsinki. All patients provided informed consent.

## Conflicts of Interest

N.B.K. has received reimbursement of meeting attendance fees and travel expenses from EISAI, lecture honoraria from the Falk Foundation and AstraZeneca, and served as an advisory board member for AstraZeneca, Roche, and Ipsen. U.E. has received honoraria for lectures from AstraZeneca, the Falk Foundation, IPSEN, and Novartis, and travel support from AstraZeneca and Biotest. She has served as an advisory board or steering committee member to AstraZeneca, Bayer, EISAI, and MSD. I.P. reports reimbursement of travel expenses from Roche and received third‐party funding for scientific research from Novartis. M.T.D. served as a speaker and/or advisory board member for AstraZeneca, Eisai, and Roche, and has received travel support from AstraZeneca and the Falk Foundation. A.K. has received lecture honoraria from Roche Pharma A.G., Eisai GmbH, Abbvie Germany A.G., Janssen‐Cilag GmbH, MSD Sharp & Dohme GmbH, Boston Scientific Corp., Fujifilm Germany, Micro‐Tech Germany, and Bayer Pharma A.G. Germany. M.P. served as a speaker and/or consultant and/or advisory board member for AstraZeneca, Bayer, Bristol‐Myers Squibb, Eisai, Ipsen, Lilly, MSD, and Roche, and received travel support from Bayer, Bristol‐Myers Squibb, Ipsen, and Roche. U.E. has received honoraria for lectures from AstraZeneca, the Falk Foundation, IPSEN and Novartis, and travel support from AstraZeneca and Biotest. She has served as an advisory board or steering committee member to AstraZeneca, Bayer, EISAI, MSD, and Roche. P.S. has served as an advisory board committee member to BMS, MSD, Eisai, and Incyte. He has received lecture honoraria from BMS, Eisai, Incyte and Janssen. He has received grants from BMS, Incyte, Falk and Chugai. J.U.M. has received honoraria for lectures, consulting activities and travel support from Roche, Eisai, AbbVie, Merz, Novo Nordisk, Ipsen, AstraZeneca, Janssen, and MSD. E.N.D.T. has served as a paid consultant for AstraZeneca, Bayer, BMS, EISAI, Eli Lilly & Co, Pfizer, IPSEN, and Roche. He has received reimbursement of meeting attendance fees and travel expenses from Arqule, AstraZeneca, BMS, Bayer, Celsion and Roche, and lecture honoraria from BMS and the Falk Foundation. He has received third‐party funding for scientific research from Arqule, AstraZeneca, BMS, Bayer, Eli Lilly, and Roche. A.G. is an advisory board or steering committee member to AbbVie, Alexion, Bayer, BMS, CSL Behring, Eisai, Falk, Gilead, Heel, Intercept, Ipsen, Merz, MSD, Novartis, Pfizer, Roche, Sanofi‐Aventis, Sequana and speaker for Advanz. F.P.R. has received honoraria for lectures, consulting activities and travel support from the Falk Foundation, AbbVie, Gilead, Ipsen, AstraZeneca, Roche, and Novartis. All other authors have nothing to declare.

## Supporting information


Data S1.

## Data Availability

The data that support the findings of this study are available on request from the corresponding author. The data are not publicly available due to privacy or ethical restrictions.
