# Supplementary material for: Therapeutic Sequences of Systemic Therapy After Atezolizumab Plus Bevacizumab for Hepatocellular Carcinoma: Real‐World Analysis of the IMMUreal Cohort
Source: Aliment Pharmacol Ther. 2025 Apr 4;61(11):1755–66. doi: 10.1111/apt.70090 (PMC12074566; doi:10.1111/apt.70090)
Supplement: Supplementary file 1 — Data S1. [file APT-61-1755-s001.pptx]

## Slide 1
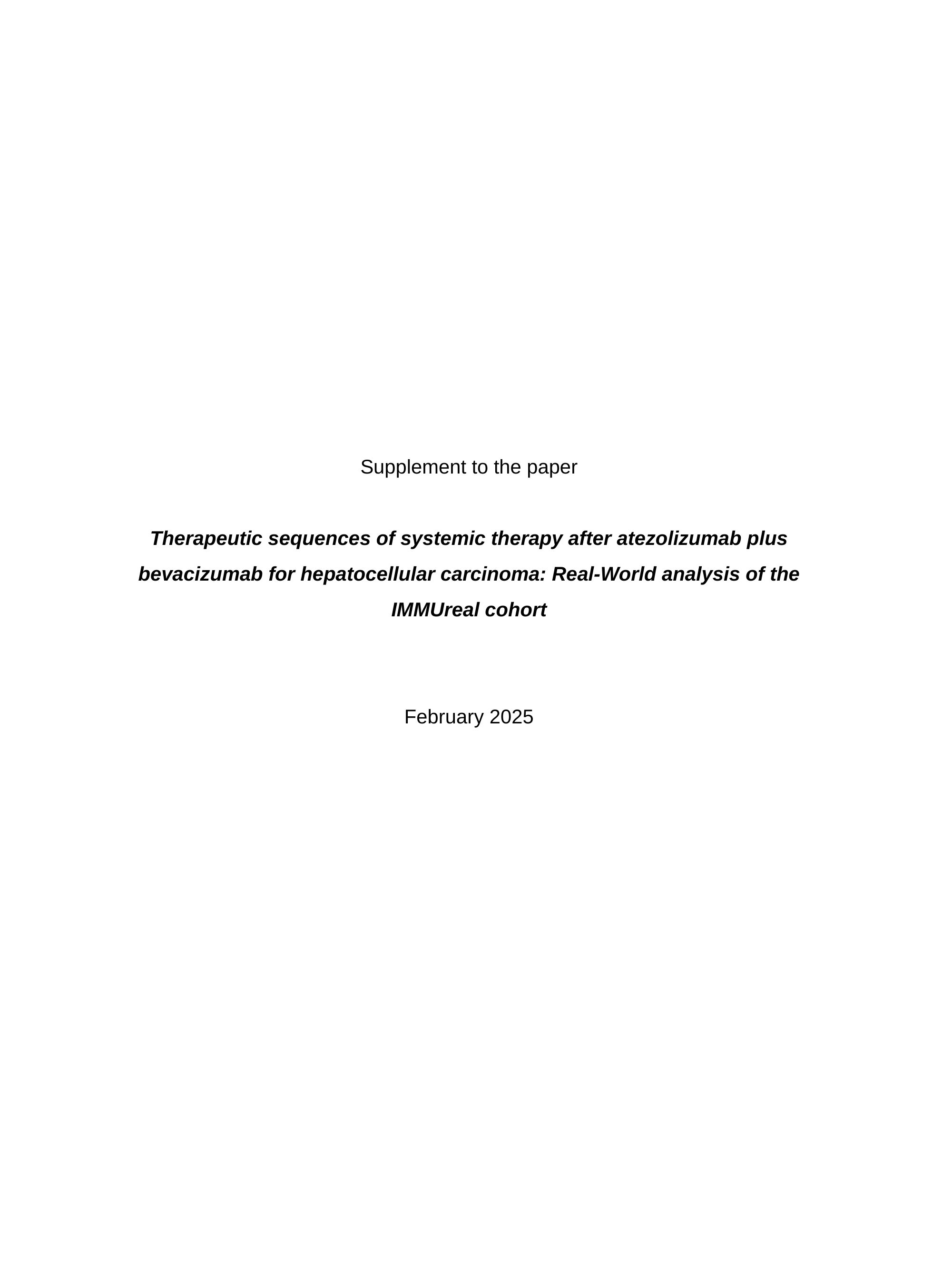

Supplement to the paper
Therapeutic sequences of systemic therapy after atezolizumab plus bevacizumab for hepatocellular carcinoma: Real-World analysis of the IMMUreal cohort
February 2025

## Slide 2
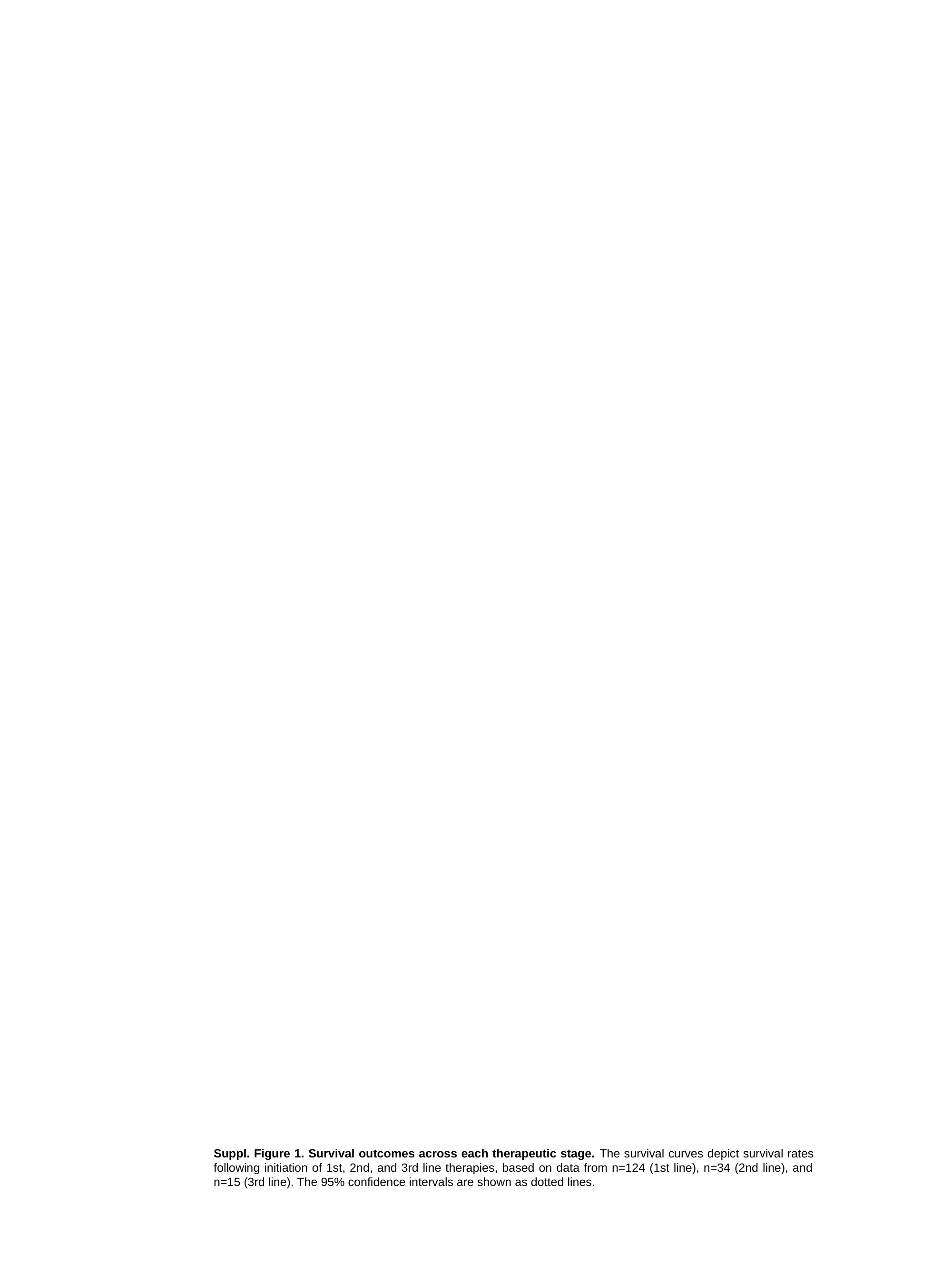

Suppl. Figure 1. Survival outcomes across each therapeutic stage. The survival curves depict survival rates following initiation of 1st, 2nd, and 3rd line therapies, based on data from n=124 (1st line), n=34 (2nd line), and n=15 (3rd line). The 95% confidence intervals are shown as dotted lines.

## Slide 3
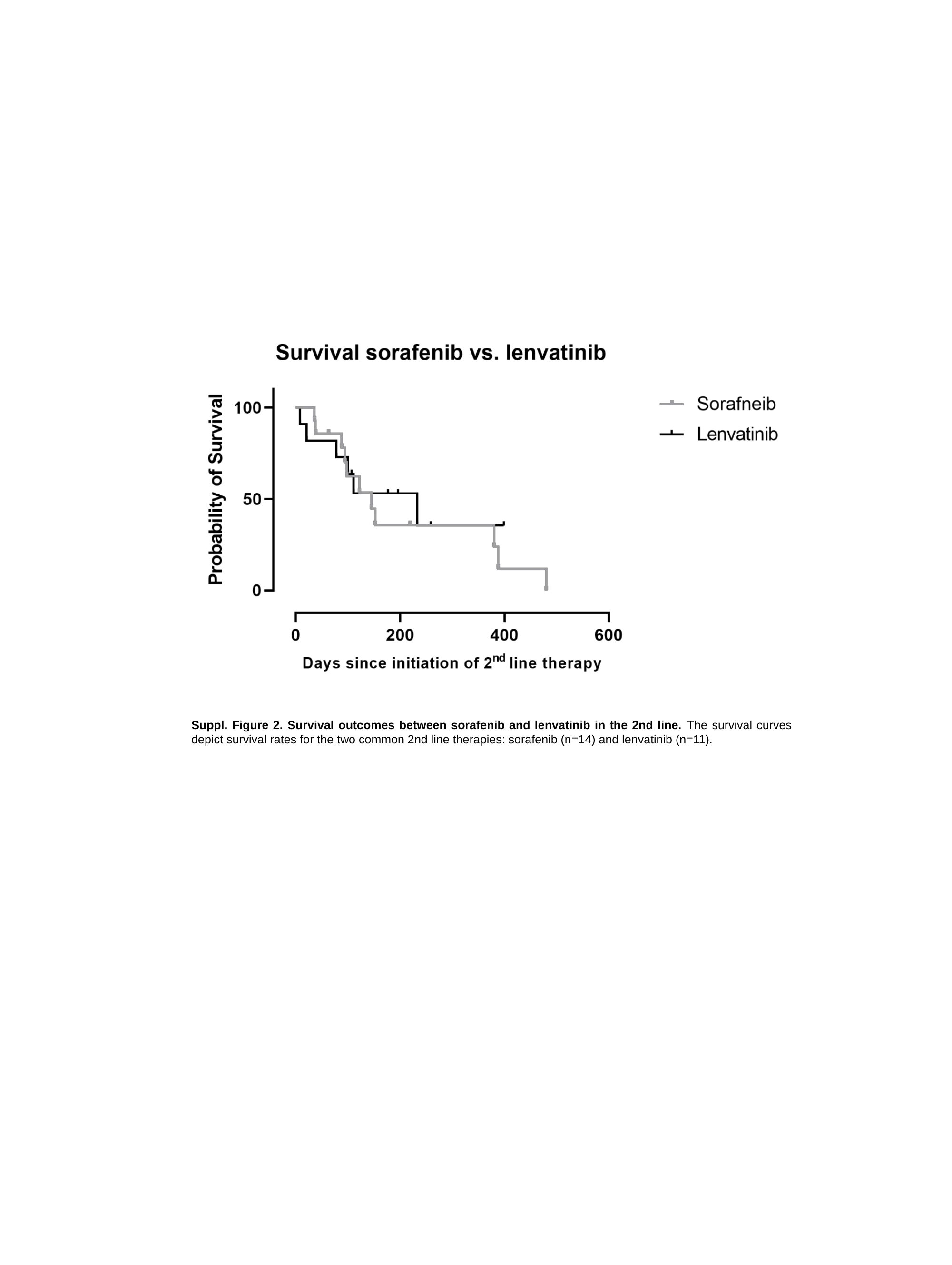

Suppl. Figure 2. Survival outcomes between sorafenib and lenvatinib in the 2nd line. The survival curves depict survival rates for the two common 2nd line therapies: sorafenib (n=14) and lenvatinib (n=11).

## Slide 4
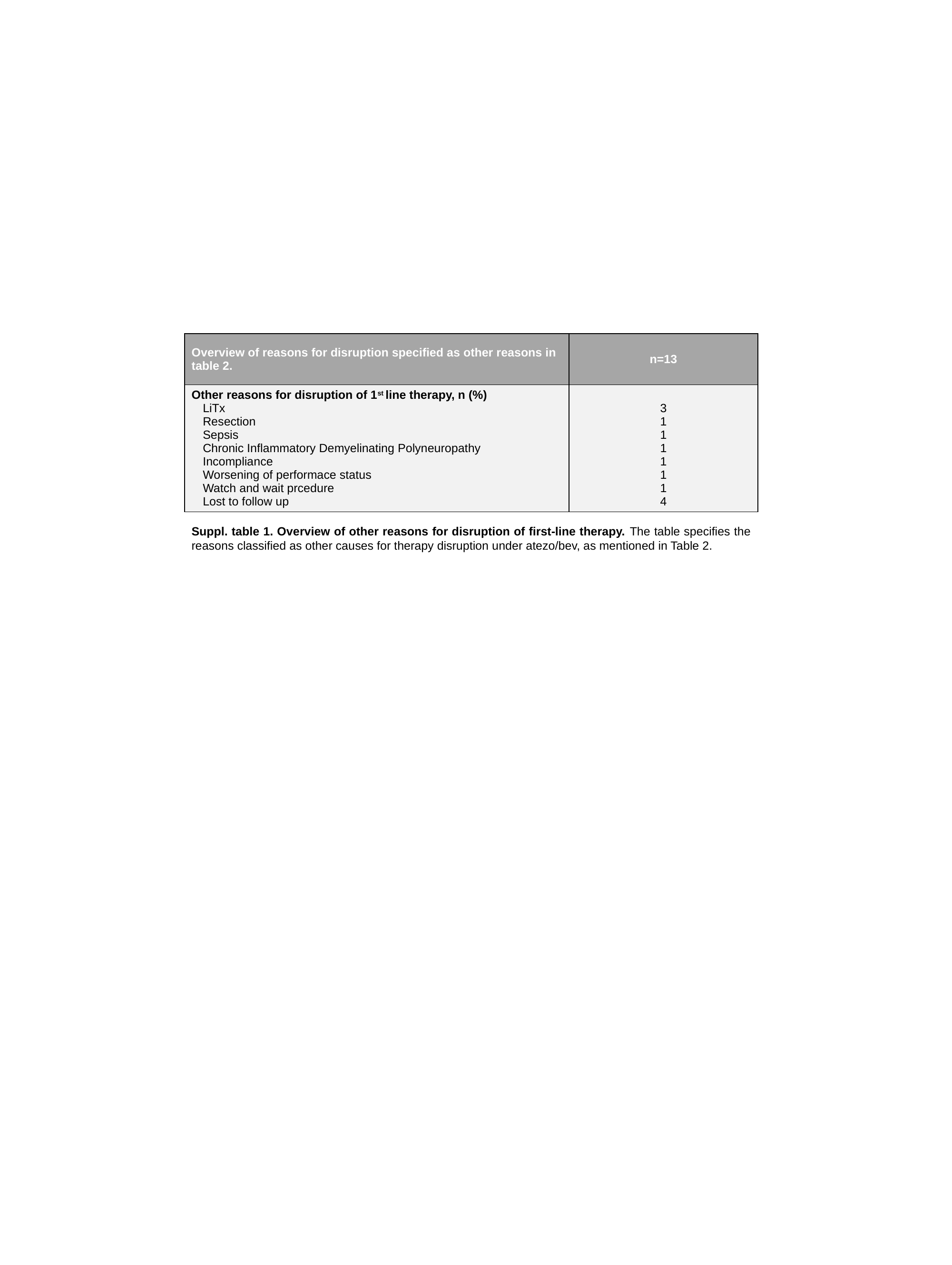

| Overview of reasons for disruption specified as other reasons in table 2. | n=13 |
| --- | --- |
| Other reasons for disruption of 1st line therapy, n (%) LiTx Resection Sepsis Chronic Inflammatory Demyelinating Polyneuropathy Incompliance Worsening of performace status Watch and wait prcedure Lost to follow up | 3 1 1 1 1 1 1 4 |
Suppl. table 1. Overview of other reasons for disruption of first-line therapy. The table specifies the reasons classified as other causes for therapy disruption under atezo/bev, as mentioned in Table 2.

## Slide 5
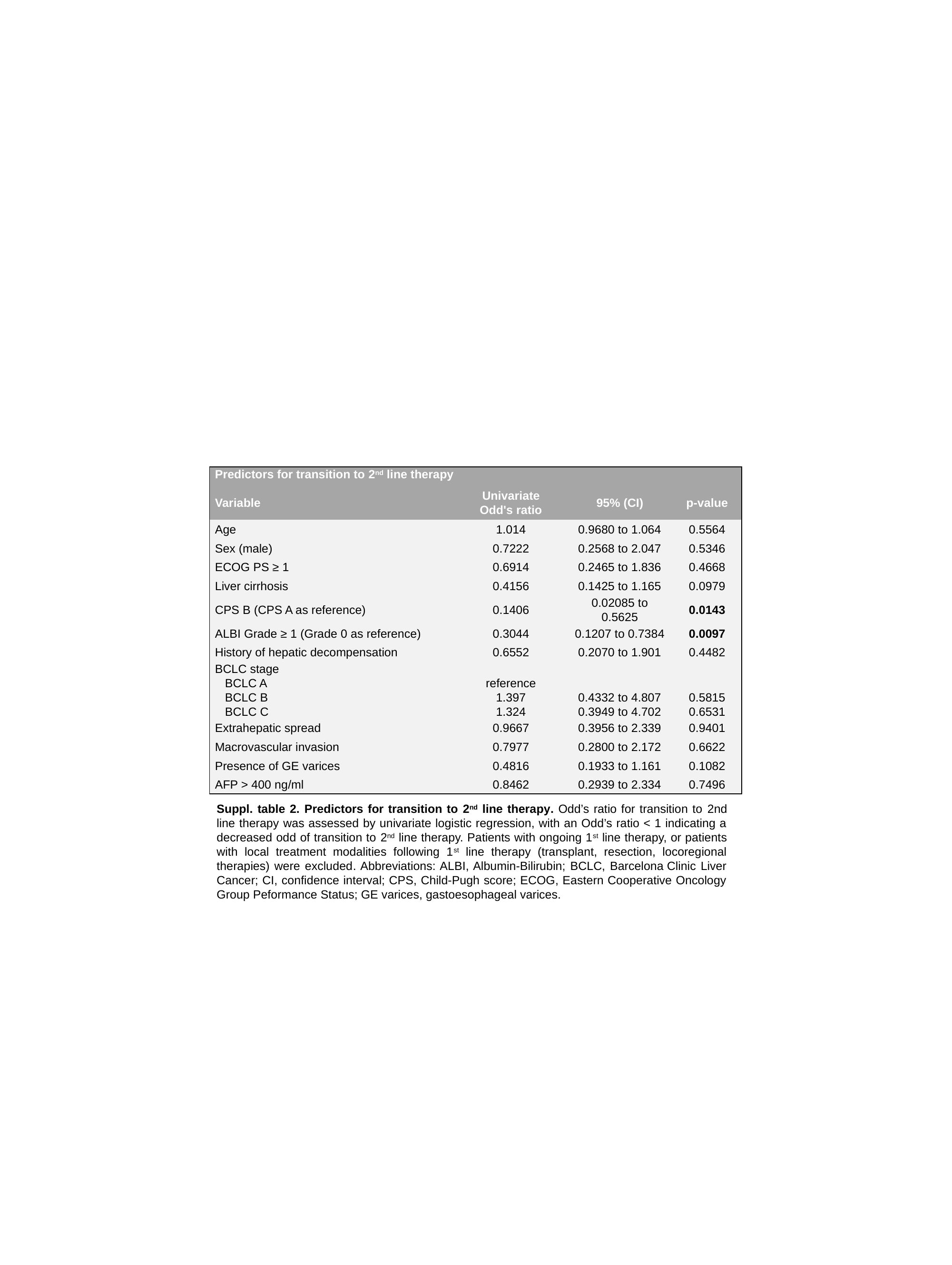

| Predictors for transition to 2nd line therapy | | | |
| --- | --- | --- | --- |
| Variable | Univariate Odd's ratio | 95% (CI) | p-value |
| Age | 1.014 | 0.9680 to 1.064 | 0.5564 |
| Sex (male) | 0.7222 | 0.2568 to 2.047 | 0.5346 |
| ECOG PS ≥ 1 | 0.6914 | 0.2465 to 1.836 | 0.4668 |
| Liver cirrhosis | 0.4156 | 0.1425 to 1.165 | 0.0979 |
| CPS B (CPS A as reference) | 0.1406 | 0.02085 to 0.5625 | 0.0143 |
| ALBI Grade ≥ 1 (Grade 0 as reference) | 0.3044 | 0.1207 to 0.7384 | 0.0097 |
| History of hepatic decompensation | 0.6552 | 0.2070 to 1.901 | 0.4482 |
| BCLC stage BCLC A BCLC B BCLC C | reference 1.397 1.324 | 0.4332 to 4.807 0.3949 to 4.702 | 0.5815 0.6531 |
| Extrahepatic spread | 0.9667 | 0.3956 to 2.339 | 0.9401 |
| Macrovascular invasion | 0.7977 | 0.2800 to 2.172 | 0.6622 |
| Presence of GE varices | 0.4816 | 0.1933 to 1.161 | 0.1082 |
| AFP > 400 ng/ml | 0.8462 | 0.2939 to 2.334 | 0.7496 |
Suppl. table 2. Predictors for transition to 2nd line therapy. Odd’s ratio for transition to 2nd line therapy was assessed by univariate logistic regression, with an Odd’s ratio < 1 indicating a decreased odd of transition to 2nd line therapy. Patients with ongoing 1st line therapy, or patients with local treatment modalities following 1st line therapy (transplant, resection, locoregional therapies) were excluded. Abbreviations: ALBI, Albumin-Bilirubin; BCLC, Barcelona Clinic Liver Cancer; CI, confidence interval; CPS, Child-Pugh score; ECOG, Eastern Cooperative Oncology Group Peformance Status; GE varices, gastoesophageal varices.
